# Burn Injury Leads to Increased Long-Term Susceptibility to Respiratory Infection in both Mouse Models and Population Studies

**DOI:** 10.1371/journal.pone.0169302

**Published:** 2017-01-09

**Authors:** Vanessa S. Fear, James H. Boyd, Suzanne Rea, Fiona M. Wood, Janine M. Duke, Mark W. Fear

**Affiliations:** 1 Tumour Immunology Group, School of Medicine and Pharmacology, University of Western Australia, Perth, Western Australia, Australia; 2 Burn Injury Research Unit, School of Surgery, University of Western Australia, Perth, Western Australia, Australia; 3 Centre for Data Linkage, Curtin University, Perth, Western Australia, Australia; 4 Burns Service of Western Australia, Fiona Stanley Hospital and Princess Margaret Hospital, Perth, Western Australia, Australia; University of South Dakota, UNITED STATES

## Abstract

**Background:**

Burn injury initiates an acute inflammatory response that subsequently drives wound repair. However, acute disruption to the immune response is also common, leading to susceptibility to sepsis and increased morbidity and mortality. Despite increased understanding of the impact of burn injury on the immune system in the acute phase, little is known about long-term consequences of burn injury on immune function. This study was established to determine whether burn injury has long-term clinical impacts on patients’ immune responses.

**Methods:**

Using a population-based retrospective longitudinal study and linked hospital morbidity and death data from Western Australia, comparative rates of hospitalisation for respiratory infections in burn patients and a non-injured comparator cohort were assessed. In addition, a mouse model of non-severe burn injury was also used in which viral respiratory infection was induced at 4 weeks post-injury using a mouse modified version of the Influenza A virus (H3NN; A/mem/71-a).

**Results and conclusions:**

The burn injured cohort contained 14893 adult patients from 1980–2012 after removal of those patients with evidence of smoke inhalation or injury to the respiratory tract. During the study follow-up study a total of 2,884 and 2,625 respiratory infection hospital admissions for the burn and uninjured cohorts, respectively, were identified. After adjusting for covariates, the burn cohort experienced significantly elevated admission rates for influenza and viral pneumonia (IRR, 95%CI: 1.73, 1.27–2.36), bacterial pneumonia (IRR, 95%CI: 2.05, 1.85–2.27) and for other types of upper and lower respiratory infections (IRR, 95% CI: 2.38, 2.09–2.71). In the mouse study an increased viral titre was observed after burn injury, accompanied by a reduced CD8 response and increased NK and NKT cells in the draining lymph nodes. This data suggests burn patients are at long-term increased risk of infection due to sustained modulation of the immune response.

## Introduction

Burns are a significant cause of mortality [1] and survivors often experience a spectrum of physical and psychological morbidity related to scarring and initial trauma [2–4]. Advances in medical management in recent years have resulted in significant declines in hospital mortality,[5, 6]. However, population-based research has shown burn survivors experience decreased long-term survival [7, 8], whilst increased long-term morbidity has been shown both in population studies and other models of burn injury [9–11].

During the acute phase after burn injury there is an increased risk of infection and sepsis-related mortality [12, 13], largely due to changes in the microflora of the skin [14], innate pro-inflammatory responses (systemic inflammatory response syndrome (SIRS)) and changes in adaptive immunity [15–17]. More recently, we have shown that the etiology of injury is important, with even non-severe burn injury leading to sustained and unique changes in immune cell profiles in a murine model [11]. However, the consequences of these changes on morbidity in the longer term are currently unknown.

Burns can also cause pulmonary dysfunction [18, 19], with the initial immune response capable of inducing pulmonary inflammation even in the absence of inhalation injury [19, 20]. The inflammatory response and resuscitative measures can both cause pulmonary damage and oedema, increasing acute post-burn susceptibility to pneumonia and acute respiratory distress syndrome [20–22].

Our previous population-based research has identified long-term cardiovascular [23] and musculoskeletal morbidity [24] as well as increased incidence of cancer [25] after both severe and minor burn injury. These results, together with animal data [11] suggests potential long-term effects of the burn on the immune system. However, to date there is a paucity of data on the consequences of immune system disruption after burn injury on long-term susceptibility and response to infection.

The objective of this study was to use population-based linked health administrative data to assess if adults hospitalized for burns had long-term respiratory infectious morbidity related to systemic effects triggered by the initial injury. We also used a mouse model of burn injury to investigate the susceptibility to infection post-burn. The population data showed increased admissions to hospital for respiratory infection in the years after discharge for burn injury, while the mouse model showed increased viral titres in a lung infection model after a burn. This work suggests sustained immune dysfunction after burn injury leads to long-term increased susceptibility to infection.

## Results

### Cohort characteristics

There were 14,893 individuals aged 18 years and older hospitalised with a first burn injury between 1980 and 2012 (who did not have a burn to the respiratory tract, head or neck, and were not ventilated during index burn admission). Our non-injury cohort comprised 61,173 individuals. The median age of the burn cohort was 35 years (interquartile range (IQR) 25–51) and 37 years (IQR 26–52) for the uninjured cohort. The mean follow up (minimum-maxiumum) for the burn and uninjured cohorts were 14.9 years (>0 to 32.5) and 15.2 years (>0 to 32.5), respectively. Baseline sociodemographic and health status variables and respective p-values for chi square tests comparing the burn and uninjured cohorts and outcomes (respiratory admissions) are presented in Table 1.

**Table 1:** Baseline demographics, pre-existing health status factors and study outcomes for the burn cohort vs. uninjured cohort.

Characteristics	No Injury N (%)	Burn injury N (%)	*P*
***Total***	61,173	14,893	
***Demographic***			
**Male**	41,305 (67.6)	10,379 (69.7)	<0.001
**Indigenous status**			
Yes	814 (1.3)	1,925 (12.9)	<0.001
**Social disadvantage quintiles** ^a^			
Quintile 1. (Most disadvantaged)	7,767 (12.7)	3,127 (21.4)	<0.001
Quintile 2.	13,854 (22.7)	4,661 (31.9)	
Quintile 3.	11,792 (19.3)	3,118 (21.4)	
Quintile 4.	11,880 (19.4)	1,813 (12.4)	
Quintile 5. (Least disadvantaged)	15,838 (25.9)	1,886 (12.9)	
**Remoteness** ^b^			
Major city	45,932 (75.1)	7,487 (51.2)	<0.001
Inner regional	6,218 (10.2)	1,590 (10.9)	
Outer regional	5,368 (8.8)	2,307 (15.8)	
Remote	2,325 (3.8)	1,658 (11.3)	
Very remote	1,288 (2.1)	1,593 (10.9)	
***Health status***			
Any comorbidity (yes)	4,165 (6.8)	2,467 (16.6)	<0.001
Prior respiratory disease admission (yes)	2,605 (4.3)	1,762 (11.8)	<0.001
Record of ventilation prior to first post-burn respiratory admission (yes)	781 (1.3)	434 (2.9)	<0.001
Ever smoked (yes)	15,979 (26.1)	6,609 (44.4)	<0.001
***Study Outcomes***			
***Total respiratory admissions***	**2,625**	**2,884**	
**Acute upper respiratory tract infections**	**353 (13.5%)**	**364 (12.6%)**	
**Influenza and pneumonia**	**1,852 (70.6%)**	**1,970 (68.3%)**	
* Influenza and viral pneumonia Pneumonia bacterial*	*127 (4*.*8%) 1*,*725 (65*.*7%)*	*109 (3*.*8%) 1*,*861 (64*.*5%)*	
**Other acute lower respiratory tract infections**	**420 (16.0%)**	**550 (19.1%)**	

^a^ SEIFA socio-economic disadvantage quintiles; missing values 1.9% burn, 0.1% no injury

^b^ ARIA+ remoteness classification; missing values 1.7% burn, 0.1% no injury

In the burn cohort, 47% (n = 6,988) had minor (<20%Total body surface area (TBSA)) burns, 2% (n = 340) had severe (≥20%TBSA) burns and for 51% (n = 7,565) the %TBSA was unspecified. For the minor burns 45% were reported as partial thickness, 25% full-thickness and 5% had records of mixed full and partial thickness injuries. For the severe burn group 27% were partial thickness, 51% full thickness and 15% recorded as mixed. Basedon the length of stay (LOS) for the index burn admission, unspecified TBSA had a median LOS (IQR: 3 days, 1–10) similar to that for minor burns 0–9% TBSA (4 days, 1–10, p<0.53) as compared to that for 10–19% TBSA (12 days, 4–19, p<0.01) or ≥ 20% TBSA (24 days, 12.5–44.5, p<0.001); suggesting unspecified TBSA burns were most likely misclassified minor burns. Overall, 18% (n = 2,627) of patients had full thickness burns, while 32% (n = 4,831) had partial thickness burns, 13% (n = 1,983) had erythema (or first degree) burns, and for 31% (n = 4,621) burn depth was unspecified

#### The burn injured cohort have significantly elevated hospital admissions for respiratory infections after discharge

During the study follow-up study a total of 2,884 and 2,625 respiratory infection hospital admissions for the burn and uninjured cohorts, respectively, were identified: influenza and viral pneumonia (109 vs. 127); bacterial pneumonia (1,861 vs. 1,725); and, other acute upper and lower respiratory infections (914 vs. 773, (Table 1)). In total, the burn and uninjured cohorts spent 46,437 and 54,586 days, respectively, in hospital with a respiratory infection diagnosis after study start. The median LOS was 4 days (IQR 2–7) and 3 days (IQR 1–7), respectively, for the burn and uninjured cohorts.

After adjusting for covariates, the burn cohort experienced significantly elevated admission rates for influenza and viral pneumonia (IRR, 95%CI: 1.73, 1.27–2.36), bacterial pneumonia (IRR, 95%CI: 2.05, 1.85–2.27) and for other types of upper and lower respiratory infections (IRR, 95% CI: 2.38, 2.09–2.71).

Summed LOS was also significantly higher for admissions for influenza and pneumonia—combined (IRR, 95%CI: 5.62, 3.33–9.49) and other respiratory infections (IRR 95%CI: 2.71, 2.04–3.06). Analysis of admission rates by burn severity showed increased admission rates for severe, minor and unspecified burn TBSA (Table 2).

**Table 2:** Adjusted incident rate ratios (IRR) and 95% confidence intervals (CI) for recurrent admissions for respiratory sub-conditions for sub cohorts defined by TBSA burn severity versus uninjured cohort.

**Respiratory sub-condition**	**Severe Burns IRR (95%CI)**	**Minor Burns IRR (95%CI)**	**Unspecified TBSA IRR (95%CI)**
**Influenza and pneumonia**	**2.69 (1.68–4.30)**	**2.65 (2.29–3.06)**	**1.82 (1.61–2.05)**
*Influenza & viral pneumonia*	*-*	*1*.*96 (1*.*21–3*.*16)*	*1*.*72 (1*.*19–2*.*47)*
*Bacterial pneumonia*	*3*.*05 (1*.*86–5*.*01)*	*2*.*69 (2*.*32–3*.*13)*	*1*.*85 (1*.*64–2*.*10)*
**Other acute upper and lower respiratory tract infections**	**3.26 (1.86–5.72)**	**2.75 (2.27–3.32)**	**2.27 (1.94–2.66)**

All models adjusted for socio-demographic (age, gender, Aboriginal status, social disadvantage, remoteness), index year and health (comorbidity, record of prior respiratory disease, ventilation, ever smoked) factors.

Assessment of the underlying temporal trends in respiratory infectious admissions during 1980–2012 for the burn and uninjured cohorts, controlling for differing lengths of follow up, identified annual declines in admissions of 6% for influenza and viral pneumonia (IRR, 95%CI: 0.94, 0.92–0.95), 6% for bacterial pneumonia (IRR, 95%CI: 0.94, 0.93–0.94) and 5% for other acute upper and lower respiratory infections (IRR, 95%CI: 0.95, 0.94–0.96).

#### Hospital admissions for acute respiratory infections are significantly elevated for the entire study period in the burn injured cohort

Survival analyses were undertaken on the burn and uninjured cohort data that excluded those with a prior respiratory admission and/or prior record of ventilation support, and additionally, those in the burn cohort who had a record of non-burn injury admission. A total number of 359 and 981 incident respiratory infection admissions were identified for the burn cohort and uninjured cohort, respectively: influenza and viral pneumonia (12 vs 40); bacterial pneumonia (127 vs. 350), other acute upper and lower respiratory infections (220 vs. 591).

Cox regression models identified significantly elevated incident admission rates for the burn cohort for acute upper and lower respiratory infections over the entire study period and for bacterial pneumonia during the first five years after discharge. The elevated incident admission rates for influenza and viral pneumonia did not reach significance, most likely due to small counts for analysis. Of the total number of first post-burn admissions for respiratory infections in the burn cohort, 153 (42.6%) could be attributed to burn injury (Table 3).

**Table 3:** Results of survival analysis for time until first or incident admission for respiratory sub-conditions, burn vs. uninjured.

	**Hazard Ratio (95% CI)**	**Attributable Risk %**	**Number of admissions attributable to burn injury**
**Influenza and viral pneumonia**			
*** 0 to 5 years after burn***	1.34 (0.67–2.68)	n.s. †	
**Pneumonia—bacterial**			
*** 0 to 5 years after burn***	2.12 (1.70–2.63)	52.8%	67
**Other acute upper and lower respiratory infections**			
*** 0 to 5 years after burn***	2.60 (1.95–3.48)	61.5%	49
*** 5 to 33 years after burn***	1.50 (1.21–1.87)	33.3%	37
***Total***			**153**

All models adjusted for socio-demographic (age at index, gender, Aboriginal status, social disadvantage, remoteness), index year, health factors (comorbidity, record of prior respiratory disease, ventilation, ever smoked).

†n.s. not significant

#### Increased viral titre observed in the lung after burn injury and subsequent influenza infection in a mouse model

**Fig 1:**
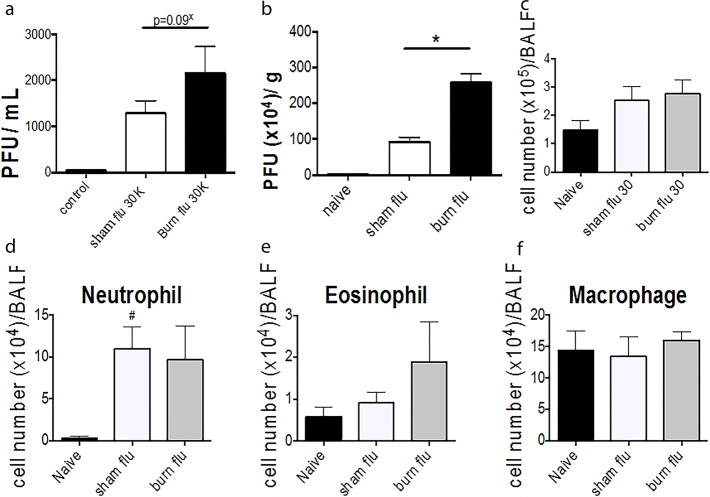
Increased viral titre in lung tissue after burn injury. Mice received a non-severe burn injury and were then challenged 4 weeks post-injury with influenza virus strain MEM71. A trend to increased viral titre was observed at 4 days post-infection in bronchalveolar lavage fluid (a) and a significant increase in viral titre observed in lung tissue (b). No difference was observed in total inflammatory cell number (c) or neutrophil (d), eosinophil (e) or macrophage (f) number between burn and sham treated animals. * p<0.05.

In light of the increased incidence of respiratory infections in the population data, we used a mouse model to investigate the immune response to Influenza A Mem/71 strain after a non-severe burn injury. At 4 weeks post injury, after recovery, we observed a trend to increased viral titre in Broncheoalveolar lavage fluid (BALF), with significantly increased viral titre in burn lung tissue. (Fig 1A and 1B). No significant difference was observed in the inflammatory infiltrate in either total cell number (Fig 1C) or neutrophil, eosinophil or macrophage infiltration (Fig 1D, 1E and 1F).

#### Diminished CD8 T cell response and elevated NK/NKT response to infection observed in draining lymph node after burn injury

**Fig 2:**
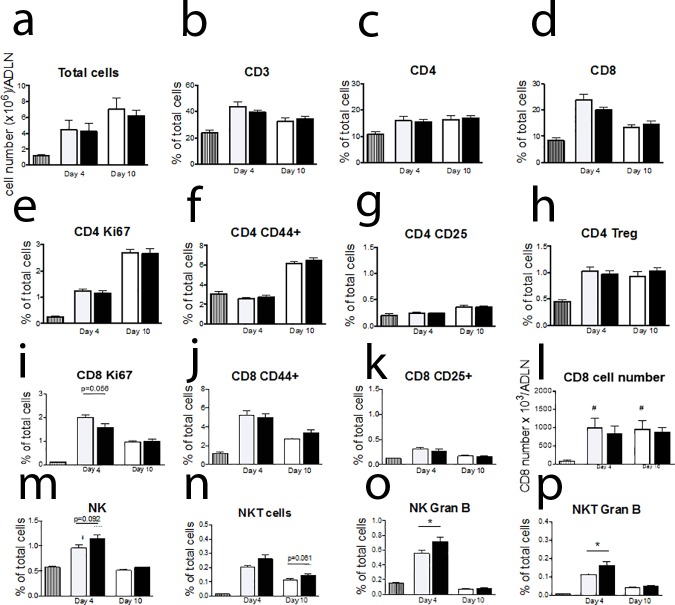
Reduced CD8 and increased NK/NKT response to viral infection in the airway draining lymph nodes after burn injury. At day 4 and day 10 post-infection no significant difference was observed in total cell number of CD3, CD4 and CD8 cell numbers (a-d). CD4 proliferation (Ki67+), antigen experience (CD44+), activation (CD25+) and CD4 Treg frequency were all not significantly different between burn and sham treatment groups (e-h). CD8 proliferation appeared to be reduced after burn injury (p = 0.056, i) with a loss of the significant increase in CD8 cells during infection compared to naïve mice seen in sham injured animals (l), but no other changes in the CD8 profile were observed (j-k). NK and NKT cell number appear to be increased at day 4 and day 10 post-infection respectively in the burn injury group (m, n, p = 0.091, p = 0.061). Gran B positive NK and NKT cells were both significantly elevated at day 4 post-infection in the burn injury group (o, p). * p<0.05.

No significant difference was observed in total lymph node cell number or the percentage frequency of CD3, CD4 or CD8 T cells (Fig 2A, 2B, 2C and 2D) in the airway draining lymph nodes (ADLN). No significant difference was observed for CD4 cell proliferation (Ki67+), antigen experience (CD44+), activation (CD25+) or CD4 Treg frequency of total cells (Fig 2E, 2F, 2G and 2H). Data indicates that the CD8 T cell response to viral infection is diminished at day 4 post-infection with a notable decrease in CD8 T cell proliferation response (Fig 2I, p = 0.056) and lack of significant increase in CD8 cell number at day 4 and 10 post-infection after burn injury compared to naïve mice (Fig 2L). Interestingly, significant changes were observed for the innate immune response to viral infection, with an enhanced NK and NKT cell frequency at day 4 and day 10 post-infection respectively (Fig 2M and 2N) and significantly increased NK and NKT Granzyme B production in the burn injury compared to the Sham (Fig 2O and 2P). This data indicates a dysfunction in the immune response to viral infection after long after burn injury wound closure, with a diminished adaptive immune response and enhanced innate immunity.

## Discussion

This study explores and quantifies long-term hospital use for respiratory infections experienced by adults who had sustained burns, after controlling for demographic and pre-existing comorbidities. The incident rate ratio analyses provided all-inclusive assessments of combined incident and prevalent admissions for respiratory diseases, and after adjustment of covariates, the burn cohort was found to have significantly higher post-burn admission rates for respiratory infections over the study period, when compared with the uninjured cohort.

Both severe and minor burns were found to have post-burn admission rates approximately 2 to 3-times greater than the uninjured cohort for respiratory infections over the study period. Burn patients experienced significantly greater first time or incident admissions for pneumonia caused by bacteria for the first 5-year post-burn period. While incident admission rates remained elevated for other types of respiratory infections over the entire study period, it was during the first 5-years post-burn that patients appeared to be at heightened risk.

The murine data also showed increased susceptibility to respiratory viral infection at 4 weeks post-injury with increased viral titre at day 4 post-infection. However there were no aditional signs of clinical infection in the burn injured mice (weight loss, ill health), most likely as the viral infection model is not severe. In this model the wound healed between 7–10 days post-injury, suggesting that even after healing there are persistent changes in the immune response. The diminished CD8 response observed in this study were also observed in our previous work suggesting CD8 activity was lower after burn injury when compared to surgical trauma of the same extent [11]. Whilst the lower response did not reach statistical significance, the absence of a significant increase in CD8 T cell numbers at either day 4 or day 10 after burn injury, in contrast to sham injured mice, strongly suggests that the CD8 response after burn injury is deficient.

Both CD8 and NK/NKT cells contribute to influenza viral clearance in man and mouse [26]. NK/NK T responses are known to function as interim effectors suppressing viral infection until CD8 T cells are activated [27]. Elevated NK/NKT cells may therefore be compensatory to dysfunctional CD8 T cell responses in burn survivors.

Previous studies have demonstrated significant changes in the immune system in the more acute phase post-burn [12, 15, 17]. However, this is the first demonstration of sustained changes with clinical impact on burn patient morbidity. With the evidence of sustained immune suppression available, this data suggests that the increased respiratory infection is likely to be driven by immune dysfunction post-burn. However, research has also shown disruption to epithelial integrity at distal uninjured sites after burn injury, and the inflammatory response has also been shown to damage lung tissue [28–30]. Therefore it is plausible that changes to lung epithelia and in particular reduced epithelial integrity or anti-microbial activity could underlie the increased incidence of respiratory infection observed.

### Strength and limitations

In the population data analysis we included socio-demographic variables, including place of residence and access to services, and health factors to examine hospital readmissions for respiratory infections. All models were adjusted for smoking status and an index of social disadvantage with demonstrated correlation with lifestyle risk factors (e.g. nutrition, smoking, alcohol, physical activity[31]). While the regression models adjusted for known confounders, given the nature of the study data, a level of residual confounding may exist. Despite smoke inhalation being involved in only a small number of burn injuries seen in WA, we nevertheless excluded any patients from the analysis that were considered to be at risk of smoke inhalation. This was done by excluding those with ICD coded burns to the head, neck or respiratory tract and those that had a record of ventilation support during the index burn admission. As such, these study findings likely reflect systemic impacts of the burn injury rather than a result of direct damage to lung tissue.

TBSA classification is included in the ICD coding systems as a supplementary code and the majority of unspecified TBSA data relate to the use of ICD9 during the period 1980–1998. Comparative analyses of hospital stay (days) for the index burn admission identified similar LOS for unspecified TBSA burns and burns of TBSA <10%. Results of negative binomial analyses comparing respiratory infectious admissions for sub burn cohorts defined by TBSA (severe, minor and unspecified burns) with the uninjured cohort revealed higher admission rates for each of the TBSA burn sub cohorts. The data were obtained from the Western Australian Data Linkage System (WADLS) which is a validated record linkage system that links all hospital and death data for the entire population of Western Australia from 1975 [32]. Regular audits of the quality and reliability of the WADLS data are undertaken by the Department of Health Western Australia. A random selection methodology was employed to identify a representative uninjured comparison cohort. Annual out-of-state migration for Western Australia is low at approximately 3% [33] and as such loss to follow-up is not an issue in this study. The use of linked hospital data limits the data to the more serious cases of respiratory infectious disease requiring hospital admission. It is therefore possible that the incidence of increased infection is much more extensive than reported here.

Further research that links clinical, pharmaceutical and/or primary care data with hospital data will enable a clearer picture of post-burn respiratory pathologies and treatment pathways.

The animal model provided additional evidence for an impact of burn injury on subsequent respiratory infection. However the use of a 4 week time-point post injury, whilst mimicking a post-discharge time-point for patients, does not necessarily establish the persistence of a longer term change. Additional tests at time-points later post-injury will be important in assessing how sustained the changes observed are, as well as the use of additional infection models to assess the impacts of the burn on immune function. The use of female mice may also impact on the generalisability of these results, as gender effects have been observed after burn injury [25, 34]. However, no impact of gender was observed in the population data in this study, suggesting the impact on long-term susceptibility to infection is not gender specific.

This data provides the first evidence of sustained immune changes after burn injury that have clinical consequences for patients. The use of hospital data may only show the ‘tip of the iceberg’ of very severe infections. Further work to understand the true extent of the increased susceptibility and the cellular pathology underlying it will be critical to improving burn patient treatment and reducing long-term morbidity in the future.

## Materials and Methods

This study forms part of the Western Australian Population-based Burn Injury Project (WAPBIP) that uses a retrospective population-based cohort design and linked health administrative data from the (WADLS [32]). The project was approved by the Human Research Ethics Committees of the University of Western Australia and the Western Australian Department of Health and project methods and analyses have been previously published [7, 23].

This study uses a de-identified extraction of all hospital morbidity records for all adults 18 years and older admitted to a hospital in Western Australia (public or private) with a first burn between 1 January 1980 and 30 June 2012. The data extraction was performed by WADLS staff. An index burn admission was defined as the first burn admission using principal or additional diagnosis data with International Classification of Diseases and Related Health (ICD) 9-CM 940–949 or ICD10-AM T20–T31 codes. Due to documented inconsistencies in both diagnosis, reporting and ICD coding of smoke inhalation and the high potential for missclassification [35], long-term effects of smoke inhalation were not included in this study. To investigate persistence of systemic effects initiated by cutaneous burn injury, analyses were conducted on the burn cohort that excluded those with a high likelihood of smoke inhalation and or direct injury to the respiratory tract (i.e. excluded those with ICD coded burns to the head, neck or respiratory tract and or had a record of ventilation support during the index burn admission).

A population-based comparison cohort was randomly selected from the Western Australian electoral roll, excluding persons with a record of an injury admission during the study period. The comparison cohort was frequency matched (~4:1) on birth year and sex of each burn injury case for each year from 1980 to 2012. For this study, the comparison of uninjured cohort comprised those members with at least one hospital admission during the study period. Data from Western Australia’s Hospital Morbidity Data System and Death Register were linked to the burn and non-injured cohorts for the period 1980–2012.

The hospital data provided ICD coded burn information (depth, site, the percentage of total body surface area (TBSA %) burned and cause). TBSA % burn severity was classified as minor (< 20% of TBSA), severe (≥ 20% TBSA) and burns with unspecified TBSA. Demographic variables included age, sex, indigenous status and postcode of residence. Indices of geographic remoteness and accessibility (Accessibility/Remoteness Index for Australia (ARIA [36]) and social disadvantage (Socio-economic Indexes for Areas (SEIFA [37]) were provided for both cohorts. Geographical remoteness was classified into five categories: major cities, inner regional, outer regional, remote and very remote. The social disadvantage index, derived from principal components analyses of over 40 national census items, was classified into quintiles (least to most-disadvantaged). The mortality data included date of death and cause of death.

A baseline comorbidity variable (any comorbidity) was derived using the Charlson comorbidity index (CCI [38]) in the hospital data with a five-year look-back period [39]. Records of prior admission (5-year look back) for any respiratory disease (ICD9-CM: 460–519; ICD10-AM: J00-J99) and ventilation (prior, during index admission, post discharge to respiratory admission) were identified. Smoking status (ever smoked–yes/no) was identified using ICD nicotine dependence codes (ICD9-CM 305.1; ICD10-AM F17.1. F17.2, Z72.0, Z86.43). The final discharge date for the index burn admission was used as the study start for follow-up for the burn cases and the respective frequency matched non-injury controls.

ICD10-AM Chapter 10 codes were used to identify principal diagnosis admissions for influenza and viral pneumonia (J10-J12), bacterial pneumonia (J13-J15) and other acute upper and lower (includes bacterial and viral) respiratory infections (J00-J06; J20-J22). The total number of principal diagnosis admissions after study start and the summed length of hospital stay (LOS) for respective respiratory admissions were used as outcomes. The admission of the first burn was not included in these outcomes. ICD9-CM codes were mapped to ICD10-AM codes [40].

Categorical and non-parametric continuous variables were compared using χ2 and Kruskal Wallis tests, respectively, and the level of significance was set at 0.05. Crude annual admission rates and LOS were calculated. Adjusted incidence rate ratios (IRR) and 95% confidence intervals (CI) were generated that compared the incident rate in the burn cohort with the incident rate in the uninjured cohort using multivariate negative binomial regression. Covariates included in the models: socio-demographic information (gender, indigenous status, age, social disadvantage, remoteness of place of residence), year of admission (to adjust for temporal referral and treatment effects) and health status (any comorbidity at baseline, ever smoked, previous respiratory disease admission, record of ventilation prior to respiratory admission). Separate analyses were conducted on subgroups defined by gender and TBSA severity (minor (<20% TBSA), severe (≥TBSA 20%) and unspecified TBSA).

Survival analyses of incident hospital use for respiratory infectious disease sub groups were conducted using multivariate Cox proportional hazards models to compare the burn cohort with the uninjured cohort. Survival analyses were conducted on the burn and uninjured cohort data that excluded those with prior records of admissions for respiratory disease and ventilation support prior to start of study follow-up, and additionally, excluding those with a non-burn injury admission (pre or post burn) in the burn cohort, to potentially exclude those with additional non-burn injury systemic effects.[11, 41] The Cox models were adjusted for covariates listed above. Preliminary analyses demonstrated nonproportionality[42] and adjusted hazard ratios (HR) and 95% confidence intervals (CI) were modelled for time periods guided by Aalen’s linear hazard models and graphs.[43]

Attributable risk percentages (AR%) were calculated as the adjusted rate ratio (HR) minus one, divided by the adjusted rate ratio (HR), multiplied by 100. [44] AR% was used to estimate the proportion of incident post-burn hospital use associated with respiratory infections where burn injury was a component cause.[41] Statistical analyses were performed using Stata version 12 (StataCorp. LP, College Station, TX, United States of America).

### Murine burn injury and infection model

#### Mice

Adult 9 week old female C57BL/6 mice were housed under pathogen free conditions with food and water provided ad libitum. Female mice were used as they can be accommodated post-injury in group housing without interference in healing of wounds of companion mice. Approval was obtained by the Telethon Kids Institute, Animal Ethics Committee (AEC#279), all experiments were performed in accordance with the National Health and Medical Research Council Australian Code of Practice for the Care and Use of Animals for Scientific Purposes.

#### Full thickness burn procedure

9 week old C57BL/6 female mice (n = 5 mice/group) received a full thickness 19-mm diameter burn wound following a previously described protocol [11]. This is equivalent to an 8% total body surface area (TBSA) injury, a non-severe injury model. Mice received burn injury 4 weeks prior to viral infection.

#### Viral infection

Influenza A/Memphis/1/71 (H3N1; A/Mem/71) is a mouse adapted strain of influenza A grown in Madin-Darby Canine Kidney Cells (MDCK) as previously described [45], and quantitated via plaque assay. Mice were lightly anaesthetised with gaseous isofluorane and then received 10^4.5^ plaque forming units (PFU) Influenza A/Mem/71 in 50μl of LPS free saline intranasally [46]

#### Broncheoalveloar lavage (BAL), cell counts and lung collection

Euthanased mice were lavaged via slow instillation/withdrawal 3 x 0.5ml saline in the lung. The cells were pelleted by centrifugation at 250g for 5 min. Total cell counts were obtained using a haemocytometer and trypan blue staining. Supernatant (broncheoalveolar lavage fluid; BALF) was collected and stored at -80°C. Cells were cytocentrifuged onto glass slides and stained with DIFF-Quik Stain (Lab Aids, Narrabeen, NSW, Australia). Light microscopy was used to determine differential cell counts.

For lungs, mice were euthanased and lung tissue collected under aseptic conditions. Lungs were weighed, homogenised, rendered a 10% homogenate in Minimal Media, and centrifuged at 250g for 5 min. Cleared lung homogenate supernatants were collected and stored at -80°C.

#### Viral titre

Cleared lung homogenate and BALF viral titre was quantified by plaque assay in MDCK cells [47]. In brief, MDCK cells were seeded into 6-well plates and grown to 95% confluence. Serial 10-fold dilutions of clarified homogenate or BALF were inoculated into each well and incubated at 37°C/5%CO_2_ for 1 h. Wells were overlayed with 0.9% agarose in L-15 media (Gibco) and plates incubated for 2 days at 37°C/5%CO_2_. Wells were fixed with 5% formaldehyde in saline, and stained with 0.5% crystal violet in methanol. Plaques were counted under an inverted light microscope.

#### Lymph node single cell preparation

Airway draining lymph nodes (ADLN) were prepared as single cell suspensions as described previously [48]. In brief, ADLN (upper paratracheal and parathymic) were harvested from individual mice, chopped with a scalpel, and digested with type IV collagenase (1.5mg/ml; Worthington) and type I DNase (0.1 mg/ml; Sigma-Aldrich) for 20 min at 37°C. All digestions and washes were performed in 5%FCS/glucose sodium potassium (GKN) buffer (11 mM D-glucose, 5.5 mM KCl, 137 mM NaCl, 25 mM Na2HPO4, 5.5 mM NaH2PO4.2 H2O) with debris and RBCs removed as described previously [48].

#### Flow cytometry

Single cell suspensions were FcR blocked (2.4G; BD Biosciences) prior to the addition of phenotyping antibodies. ADLN T cell innate cell populations were identified using the antibodies CD3, CD4, CD8, CD25, CD44, TCTg/d, and NK1.1 (PK136). Labeling was performed in GKN buffer containing 0.2% BSA for 30 min on ice. A FOXP3 intracellular staining kit (eBiosciences, San Diego, CA) was used to determin intracellular Granzyme B, Ki67 (BD Biosciences), and FOXP3 levels. All antibodies were used as direct conjugates to FITC, Phycoerythrin (PE), PE-Cy7, allophycocyanin (APC), APC-Cy7, Brilliant Violet 421, Brilliant Violet 650, Brilliant Violet 711, Brilliant Violet 786, PE-CF594. (BD Biosciences, San Jose, CA) as previously described [49]. Appropriately matched IgG isotype controls and cytometer compensation settings adjusted using single-stained controls, and Fluoresence Minus One (FMO) were used for each experiment. Samples were collected using an LSRFortessa flow cytometer (BD Biosciences) and analysed using Flowjo software (TreeStar).

#### Statistical analysis

All results were analysed using Prism 6 (Graphpad software). Differences between groups were compared using Kruskal-Wallis test with Dunn’s test for multiple comparisons.
